# Surgical Management of Endodontic Failure and Dental Neglect: A Pediatric Case Report

**DOI:** 10.7759/cureus.90854

**Published:** 2025-08-24

**Authors:** Mridula Goswami, Farheen Sultan, Rimshheanam Rimshheanam

**Affiliations:** 1 Pediatric and Preventive Dentistry, Maulana Azad Institute of Dental Sciences, New Delhi, IND

**Keywords:** apicoectomy, dental operating microscope (dom), endodontic treatment, mineral trioxide aggregates, non-surgical endodontic treatment

## Abstract

The ultimate goal of an endodontic treatment is to eliminate infection, thoroughly clean and disinfect the root canal system, and prevent reinfection. Although endodontic procedures generally demonstrate high success rates, failures may still occur due to factors such as inadequate obturation or overextension of root-filling materials. Gutta-percha extrusion beyond the apex can lead to persistent inflammation and compromise the healing. This case report presents a 12-year-old girl with a two-month history of continuous pain and swelling in the maxillary anterior region. The patient had a history of dental trauma and previous root canal treatment and retreatment for tooth 21. Clinical and radiographic findings revealed periapical radiolucency with incomplete and over-extruded obturated material. An attempt to remove the extruded material using Hedstrom files under a Dental Operating Microscope (DOM) was unsuccessful, and persistent symptoms necessitated surgical intervention. This case presents the clinical challenges associated with an over-extended obturation and the limitations of nonsurgical retreatment in managing such conditions. Surgical endodontics, guided by American Association of Endodontists (AAE) protocols, can effectively manage persistent symptoms associated with over-extruded filling material. Timely intervention and adherence to clinical protocols are critical in ensuring successful long-term outcomes.

## Introduction

Endodontic treatment encompasses the etiology, diagnosis, prevention, and treatment of diseases and injuries affecting the dental pulp and the associated periradicular tissues. Root canal treatment, a common endodontic procedure, involves the removal of inflamed or necrotic pulp tissue, followed by chemo-mechanical debridement, disinfection, and obturation of the root canal system using biocompatible materials to prevent reinfection and promote periapical healing [1]. The long-term survival rate of teeth following primary root canal treatment (RCT) ranges from 86% to 93% over two to 10 years, with a reported success rate exceeding 85% [2]. However, treatment failure and symptom recurrence occur in approximately 10% to 15% of the cases [3]. When failure occurs, non-surgical root canal retreatment is typically the first-line approach. This involves the complete removal of the existing root canal filling material to enable effective disinfection and resolution of persistent infection [4]. In contemporary endodontics, the use of a Dental Operating Microscope (DOM) has significantly enhanced visualization and precision during retreatment [5]. In certain cases, complications such as overextended obturation or fragments of gutta-percha beyond the apex can pose serious challenges to retreatment. The complications associated with over-extruded gutta-percha include persistent periapical inflammation and pain, foreign body reaction leading to granuloma or fibrosis, and compromised apical seal [6]. These adverse effects not only hinder periapical healing but also complicate future interventions. Therefore, strict adherence to the American Association of Endodontists (AAE) guidelines is crucial.

This case report describes the endodontic retreatment of a permanent maxillary central incisor in a pediatric patient, which was complicated by a previously ill-filled root canal and overextended gutta-percha. The case presents parental negligence in bringing the child to the clinician to providing comprehensive management. Hence, this case highlights the importance of timely and appropriate dental care and emphasizes the significance of retreatment cases with a focus on patient welfare.

## Case presentation

A 12-year-old female patient reported with pain and swelling in the maxillary anterior region for the past two months. The patient had no relevant medical history, with no reported systemic illnesses or ongoing medications. The patient gave a history of accidental trauma in the maxillofacial region while playing two years back. Approximately two weeks after this incident, she underwent root canal treatment (RCT) for tooth 21 at a local dental clinic. Even after the completion of RCT, the patient kept experiencing intermittent pain in the same region for approximately nine months. This was dental neglect on behalf of the child’s family. After nine months, the same dentist was consulted, and she underwent re-treatment of tooth 21.

**Figure 1:**
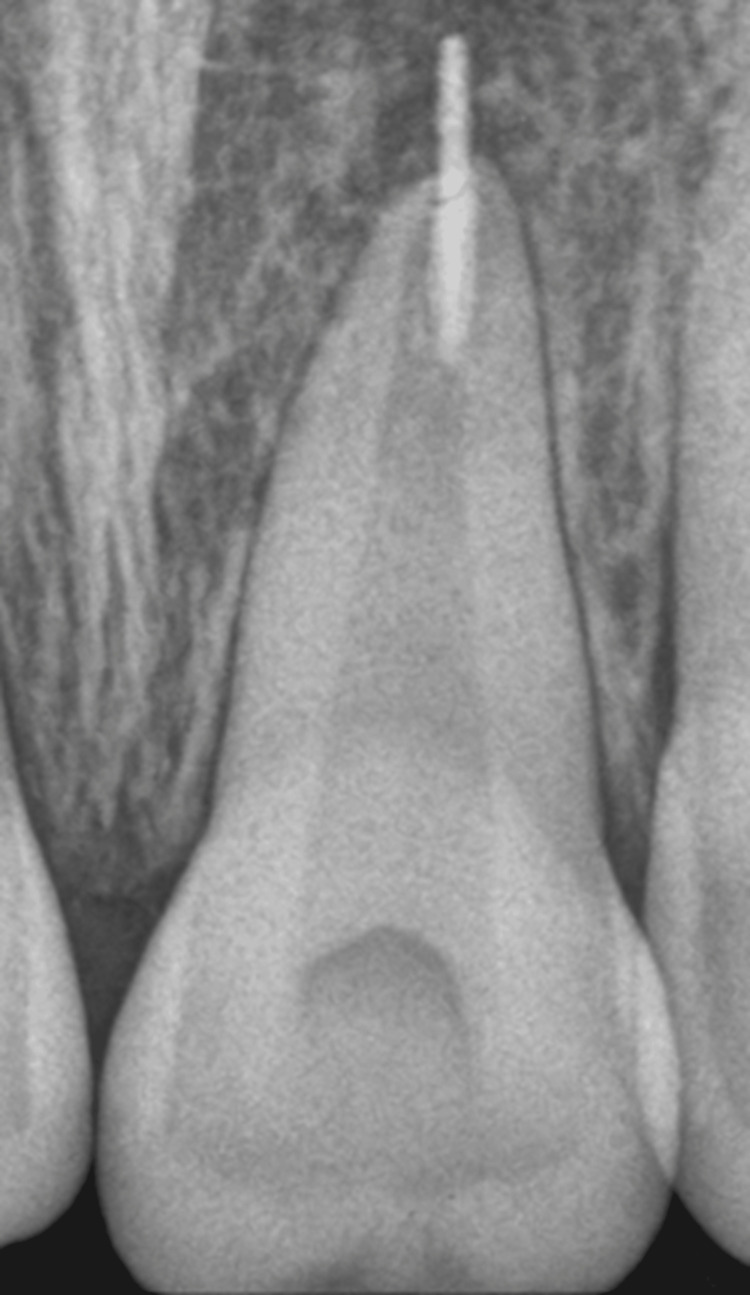
Pre-operative radiovisiography (RVG) of tooth 21 showing over-extruded gutta-percha with incomplete obturation.

The patient reported to us without any past clinical and radiographic records, and only the case history was our basis to start this case. Clinical examination revealed tenderness on percussion in relation to the permanent maxillary left central incisor and radiographic evaluation showed periapical radiolucency with Periapical Index (PAI) score of 4 with incomplete obturation and over-extruded gutta percha in tooth 21 (Figure 1).

**Figure 2:**
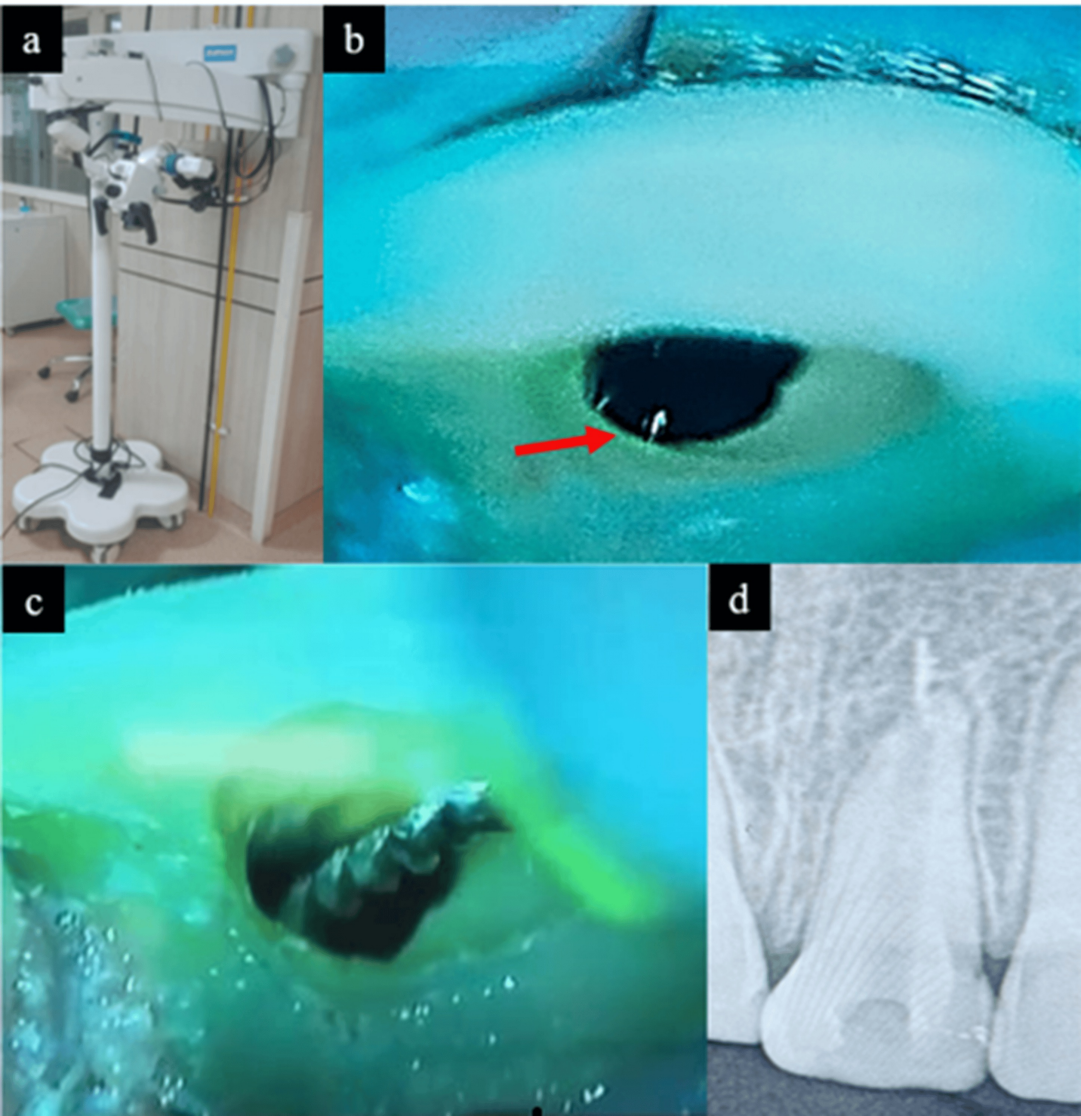
(a) Dental Operating Microscope (DOM); (b) Red arrow indicates a shiny spot like reflection of foreign material (gutta percha (GP)) under DOM; (c) Attempt to retrieve GP using H-file under DOM; (d) Radiovisiography (RVG) of tooth 21, showing intracanal GP (4-5 mm) removed, over-extruded GP beyond apex not removed.

Upon reviewing the patient's history, and clinical and radiographic findings, the decision was made to proceed with a conservative treatment approach. After obtaining the informed consent, an attempt to remove the extruded gutta-percha using Hedstrom files (H-file) was performed under the DOM (Figure 2a-c), but only intracanal fragments of gutta-percha were removed (Figure 2d).

**Figure 3:**
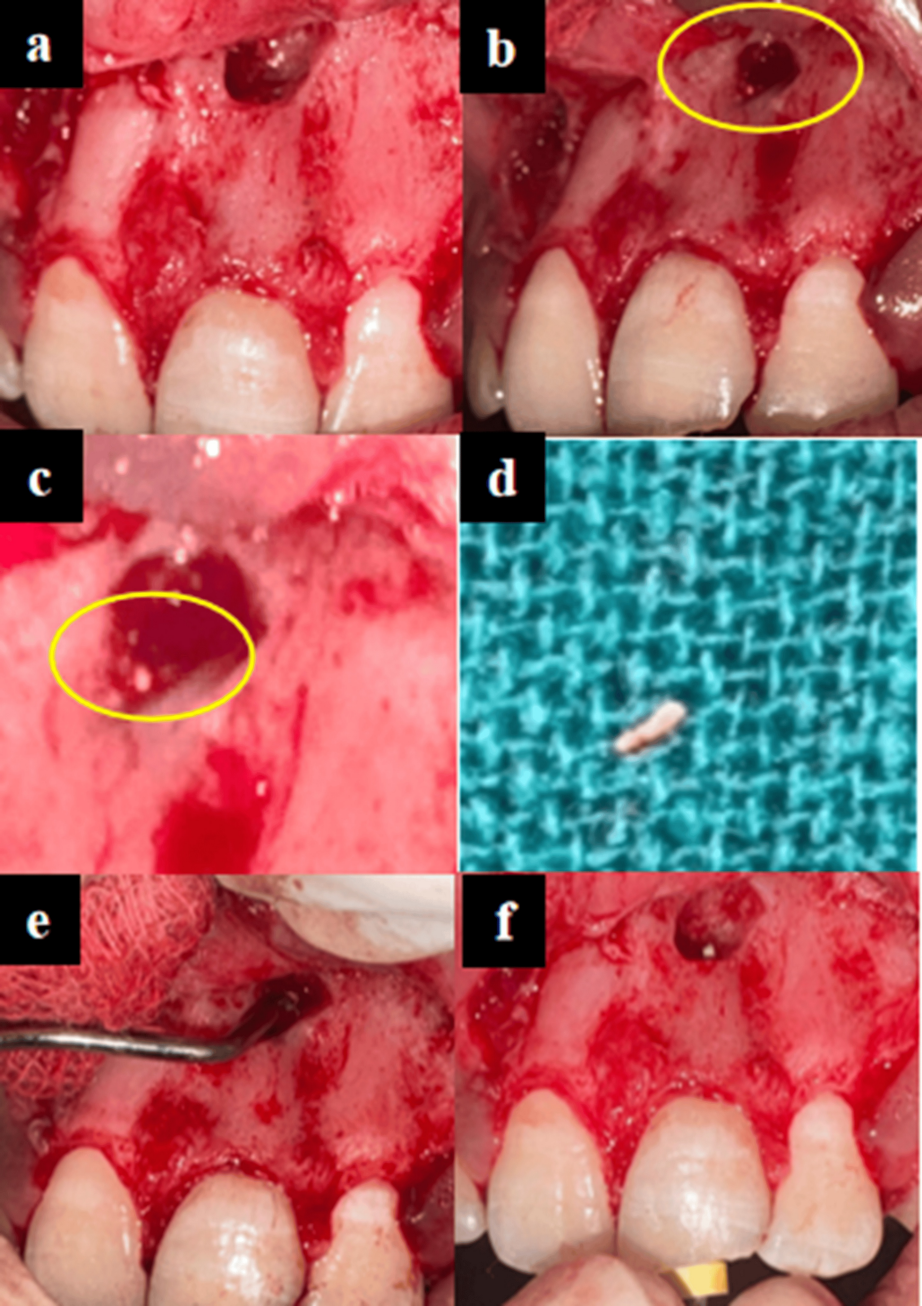
(a) Mucoperiosteal flap raised, bone guttering exposing peri-apical area of tooth 21; (b) tip of extruded gutta-percha seen; (c) magnification of figure (b); (d) retrieved GP fragment; (e) debridement of the socket; (f) root resection done and intra-canal chemomechanical debridement

The gutta-percha fragment beyond the apex could not be retrieved, and the patient continued to experience persistent pain and discomfort in the same tooth region. Hence, apicoectomy was planned as per the AAE guidelines [1]. Before surgery, baseline hematological investigations including complete blood count (CBC), bleeding time (BT), clotting time (CT), and prothrombin time/international normalized ratio (PT/INR) were conducted [7]. All values were within normal limits, with the INR measured at 1.0. Parental informed consent and patient assent were duly obtained prior to the procedure. Under local anesthesia, a trapezoidal flap was reflected with a horizontal incision along the gingival sulcus and two vertical releasing incisions at an angle of approximately 45-degree at the distal aspects of 11 and 22, and full thickness mucoperiosteal flap was elevated, ensuring the flap extends beyond the area of the root apex to allow adequate access to the surgical site. Bone guttering was done using a low-speed round bur (Diamond Bur ISO size 4; Prime Dental Products, New Delhi, India) to allow access to the apical region of tooth 21 (Figure 3a). After exposing the apex, the fragment of GP was removed using a micro tweezer and 2-3 mm of the root tip was resected using a small round bur and angled micro-handpiece (Figure 3b-d). Curettage of the surrounding periapical tissue was performed to ensure the elimination of remaining infected tissue and debris (Figure 3e). The resected root end was restored with orthograde filling of mineral trioxide aggregate (MTA Angelus, Londrina, Brazil) to achieve an apical seal. Retrograde filling was omitted to avoid excessive apical exposure, favouring a conservative approach to minimize surgical complication. This approach aligns with the principles of apexification, wherein an apical barrier is established to facilitate periapical healing and prevent reinfection. The access cavity was temporarily sealed with restorative material after placing a moistened cotton pellet in the canal (Figure 3f).

**Figure 4:**
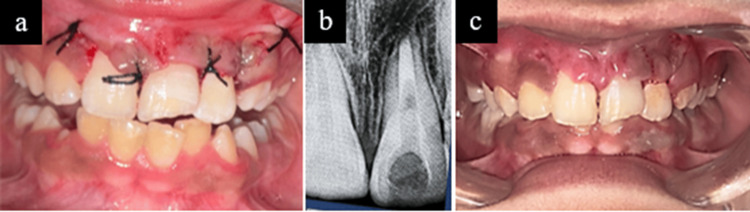
(a) Immediate closure with suturing; (b) immediate post-operative radiovisiography (RVG) showing complete removal of extruded gutta-percha and mineral trioxide aggregate (MTA) filling; (c) one-week follow-up after suture removal

After carefully repositioning the flap, 3-0 silk sutures (Ethicon, Johnson & Johnson, Cincinnati, OH, USA) in an interrupted manner were placed, followed by a post-operative radiograph (Figure 4a,b). The patient was prescribed analgesics and antibiotics to minimize the risk of postoperative infection [8]. At the one-week follow-up, the patient presented with signs of poor oral hygiene and localized gingivitis, likely due to apprehension about brushing in the surgical area postoperatively. After careful evaluations, sutures were removed and maintaining oral hygiene and plaque control during the healing phase was reinforced to the patient (Figure 4c).

**Figure 5:**
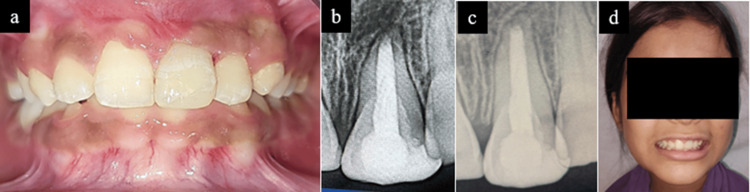
(a) Two-week follow-up showing gingival healing and composite restoration of tooth 21; (b) Two-week radiovisiography (RVG) of tooth 21 showing obturation and composite restoration; (c) six-month follow-up RVG showing radiographic success; (d) six-month follow-up showing extra-oral view and patient’s smile achieved.

At two-week follow-up, obturation was completed with gutta-percha, followed by composite restoration, as the patient was asymptomatic and showed notable improvement in gingival health and oral hygiene status (Figure 5a,b). The patient was monitored at one, three, and six months. At the six-month follow-up, radiographic examination showed evidence of periapical healing with reformation of the lamina dura. The PAI score improved from 4 to 2. Clinically, the patient was asymptomatic with a Visual Analog Scale (VAS) pain score of 0. Complete root formation with a conical apex was observed, indicating a favourable prognosis with satisfactory aesthetic, functional rehabilitation, improved smile aesthetics, and overall improvement in quality of life (Figure 5c,d).

## Discussion

Endodontic microsurgery employs high magnification and illumination provided by the DOM, along with specialized microsurgical instruments, to improve precision and enhance clinical outcomes [9]. The DOM enhances visualization and precision during the retrieval of gutta-percha from root canals, enabling more conservative and accurate removal, particularly in complex canal anatomies [10]. In the present case, an attempt was made to retrieve the extruded material through the root canal re-treatment (non-surgically) under DOM magnification. On magnification, a shiny spot at the end of the root canal was seen, which was assumed to be a reflection from foreign material, but the attempt was not successful. As a result, surgical removal of the extruded material became necessary.

Apicoectomy involves the surgical resection of the root apex and the surrounding infected tissues, providing direct access to the apical area where the infection resides. Table 1 outlines the indications and contraindications for apicoectomy [11].

**Table 1:** Indications and contraindications of apicoectomy. This table is an original compilation of information from various established resources [11].

Indications	Contraindications
Extruded filling materials presenting with clinical and radiographic signs of persistent apical periodontitis may require surgical management.	Vertical root fracture (VRF)
Irreversibly obstructed root canals may necessitate periapical surgical management.	Tooth with poor periodontal health
Challenges in maintaining adequate oral hygiene due to limited access in the affected region.	Patient with a compromised medical history
Significant root exposure resulting from dehiscence.	Un-cooperative patient
Unfavorable positioning of roots in adjacent teeth.	
Substantial vertical bone loss in multirooted teeth that is limited to a single root.	

During an apicoectomy, the root end is resected to a depth of 3-4 mm to remove any infected tissue and expose healthy dentin [12]. The root resection should ideally be performed at a right angle to its long axis [13]. In cases where the root is in close proximity to structures like the maxillary sinus or mandibular canal, gradual grinding is preferred over cutting to minimize the risk of displacing the root tip [12].

Various materials have historically been used for root-end filling, including gold, amalgam, resin composites, and glass ionomer cement [14]. In recent years, advanced bioceramic materials such as MTA, Biodentine, and EndoSequence have gained prominence due to their superior physicochemical and biological properties [15]. Among these, MTA is considered the gold standard due to its high biocompatibility, effective sealing properties, and capacity to promote cementum formation and regeneration of periapical tissues [16]. Clinical studies have reported high success rates with MTA in endodontic surgeries, although differences with other materials are not always statistically significant [17]. Healing is typically assessed both clinically and radiographically at approximately one year following surgery [18]. However, smaller periapical defects (less than 5 mm in diameter) may demonstrate evidence of clinical and radiographic healing within a few months. (Table 2) [19].

**Table 2:** Clinical and radiographic evaluation criteria. Radiographic evaluation criteria [18]. This table is an original compilation of information from various established resources [19].

Clinical Evaluation Criteria	Radiographic Evaluation Criteria
Absence of pain	Complete healing
Absence of sinus tract	Incomplete healing (“formation of scar tissue”)
Absence of swelling	Uncertain healing (partial radiographic healing after surgery)
Absence of apico-marginal communication	Evidence of unsatisfactory healing (persistence or enlargement of postsurgical radiolucency)
Absence of tenderness to percussion or palpation	

Failed endodontic treatment of anterior teeth can cause aesthetic issues such as discoloration and swelling, leading to psychosocial impacts including embarrassment, social withdrawal, and reduced self-esteem. Differences between adults and children are evident in certain aspects of complaints. Children are often not able to clearly explain the kind of pain and discomfort they experience, and they are usually less expressive about aesthetic concerns. Therefore, the role of parents and guardians is extremely important in understanding their children and taking them to the clinician for timely treatment. This case highlights the importance of regular follow-ups and proper monitoring after treatment to avoid dental complications.

## Conclusions

Endodontic complications in young permanent teeth require prompt diagnosis and timely intervention to prevent long-term consequences such as tooth loss, which can have significant functional and aesthetic implications. Non-surgical retreatment was initially attempted, but persistent symptoms and the inability to retrieve the extruded gutta-percha beyond the apex necessitated a surgical approach (apicoectomy). The use of advanced techniques, such as DOM, played a critical role in improving visibility and precision during treatment, reflecting modern endodontic standards. Apicoectomy, though a long-standing treatment modality, remains a highly effective modality in cases where non-surgical treatment fails, particularly for eliminating periapical pathology and preserving the natural tooth. MTA was used as the root-end filling material due to its superior sealing ability and biocompatibility, aligning with current best practices in endodontic surgery. This case highlights that a combination of contemporary materials and the use of the latest technological equipment along with sound clinical judgment, is essential for managing complex endodontic failures and achieving long-term functional success in pediatric patients.
